# Modelling pulmonary microthrombosis coupled to metastasis: distinct effects of thrombogenesis on tumorigenesis

**DOI:** 10.1242/bio.024653

**Published:** 2017-03-16

**Authors:** Colin E. Evans, Asis Palazon, Jingwei Sim, Petros A. Tyrakis, Alice Prodger, Xiao Lu, Saria Chan, Pär-Ola Bendahl, Mattias Belting, Love Von Euler, Helene Rundqvist, Randall S. Johnson, Cristina Branco

**Affiliations:** 1 Department of Physiology, Development and Neuroscience, University of Cambridge, Cambridge, CB2 3EG, UK; 2 British Heart Foundation Centre of Research Excellence, University of Cambridge, Cambridge, CB2 3EG, UK; 3 Department of Clinical Sciences, Lund University, Lund, SE-221 00, Sweden; 4 Department of Cell and Molecular Biology, Karolinska Institutet, Stockholm SE-171 77, Sweden

**Keywords:** HIF, Hypoxia, Thrombosis, Metastasis, Microvascular

## Abstract

Thrombosis can cause localized ischemia and tissue hypoxia, and both of these are linked to cancer metastasis. Vascular micro-occlusion can occur as a result of arrest of circulating tumour cells in small capillaries, giving rise to microthrombotic events that affect flow, creating localized hypoxic regions. To better understand the association between metastasis and thrombotic events, we generated an experimental strategy whereby we modelled the effect of microvascular occlusion in metastatic efficiency by using inert microbeads to obstruct lung microvasculature before, during and after intravenous tumour cell injection. We found that controlled induction of a specific number of these microthrombotic insults in the lungs caused an increase in expression of the hypoxia-inducible transcription factors (HIFs), a pro-angiogenic and pro-tumorigenic environment, as well as an increase in myeloid cell infiltration. Induction of pulmonary microthrombosis prior to introduction of tumour cells to the lungs had no effect on tumorigenic success, but thrombosis at the time of tumour cell seeding increased number and size of tumours in the lung, and this effect was strikingly more pronounced when the micro-occlusion occurred on the day following introduction of tumour cells. The tumorigenic effect of microbead treatment was seen even when thrombosis was induced five days after tumour cell injection. We also found positive correlations between thrombotic factors and expression of HIF2α in human tumours. The model system described here demonstrates the importance of thrombotic insult in metastatic success and can be used to improve understanding of thrombosis-associated tumorigenesis and its treatment.

## Abstract (precis)

**Summary:** Induction of pulmonary microthrombosis by three distinct methods enhances HIF-a expression and tumour formation; increases in tumorigenesis that are induced by these thrombotic insults occur in a time- and mode-dependent manner.

## INTRODUCTION

There is extensive evidence that cancer is a thrombotic disease (Blom et al., 2005; Douketis et al., 2009; Sorensen et al., 2012). Significant levels of thrombosis accompany many cancers, notably lung cancer, and cancer treatments such as cytotoxic chemotherapy are themselves thrombotic (Blom et al., 2005). Since the stage of furthest progression of most cancers is its dissemination to distant organs, or metastases, and since the lung is one of the most common and devastating areas for secondary tumour establishment, it is important to understand how thrombotic events could lead to, or even promote, metastatic success.

Thrombotic events in cancer include thrombosis and thromboembolisms, which may be caused by a range of factors, including tumour-induced changes in plasma coagulants, inflammation (and the consequent activation of the endothelium leading to increased adhesion), along with vessel obstruction, whether by tumour-derived cell debris or circulating tumour cells (Varki, 2007). As the pulmonary capillary bed is so extensive and sustains such a high rate of circulatory throughput, many of these effects are necessarily concentrated there. Thromboembolism is a common cause of death in cancer patients, and both thrombosis and thromboembolism are independent indicators of this condition (Douketis et al., 2009; Sorensen et al., 2012); this association is substantiated by evidence that that anti-coagulants protect against pulmonary metastasis, while thrombotic agents have the opposite effect (Belting, 2014; Langer et al., 2006; Varki, 2007; Wenzel et al., 2010). There is, however, a lack of experimental models that facilitate elucidation of the mechanisms by which microvascular occlusion and thrombosis promote metastasis.

The primary aim of this study was to develop a mouse model of thrombosis-associated metastasis in the pulmonary vasculature, and to determine how microvascular occlusion affects pulmonary tumour formation. We show here that pulmonary microthrombosis enhances tumour formation, and that it does so in a manner that is dependent on the time at which such insult is induced with respect to the introduction of tumour cells. The model systems presented in this study provide an experimental means of investigating thrombosis-associated tumour spread and its potential treatment.

## RESULTS

### Microbead-induced pulmonary microvascular occlusion induces acute microthrombosis

During tumorigenesis, microthrombi can arise in a number of ways, such as from microvascular occlusion by tumour cell aggregates, or fibrin formation as a result of tumour-increased clotting mechanisms (Varki, 2007). To investigate the relationships between thrombosis, hypoxic response, and metastatic success, we developed a method for modelling ischemic/thrombotic events, one that removed the biological component of the event and its inherent variability. We injected fluorescently-tagged polystyrene microbeads with a mean diameter of 15 μm into the tail vein of adult mice. As pulmonary capillaries in rodents have been shown to have a mean diameter of approximately 6 μm, with a range of between 3-11 μm (Short et al., 1996), a bead this size was expected to pass through the venous circulation and pre-capillary pulmonary circulation to cause a direct blockage of capillary flow; this method can thus allow direct determination of the effect of timed, coordinated ischemic events on the fate of tumour cells in the venous circulation.

**Fig. 1.**
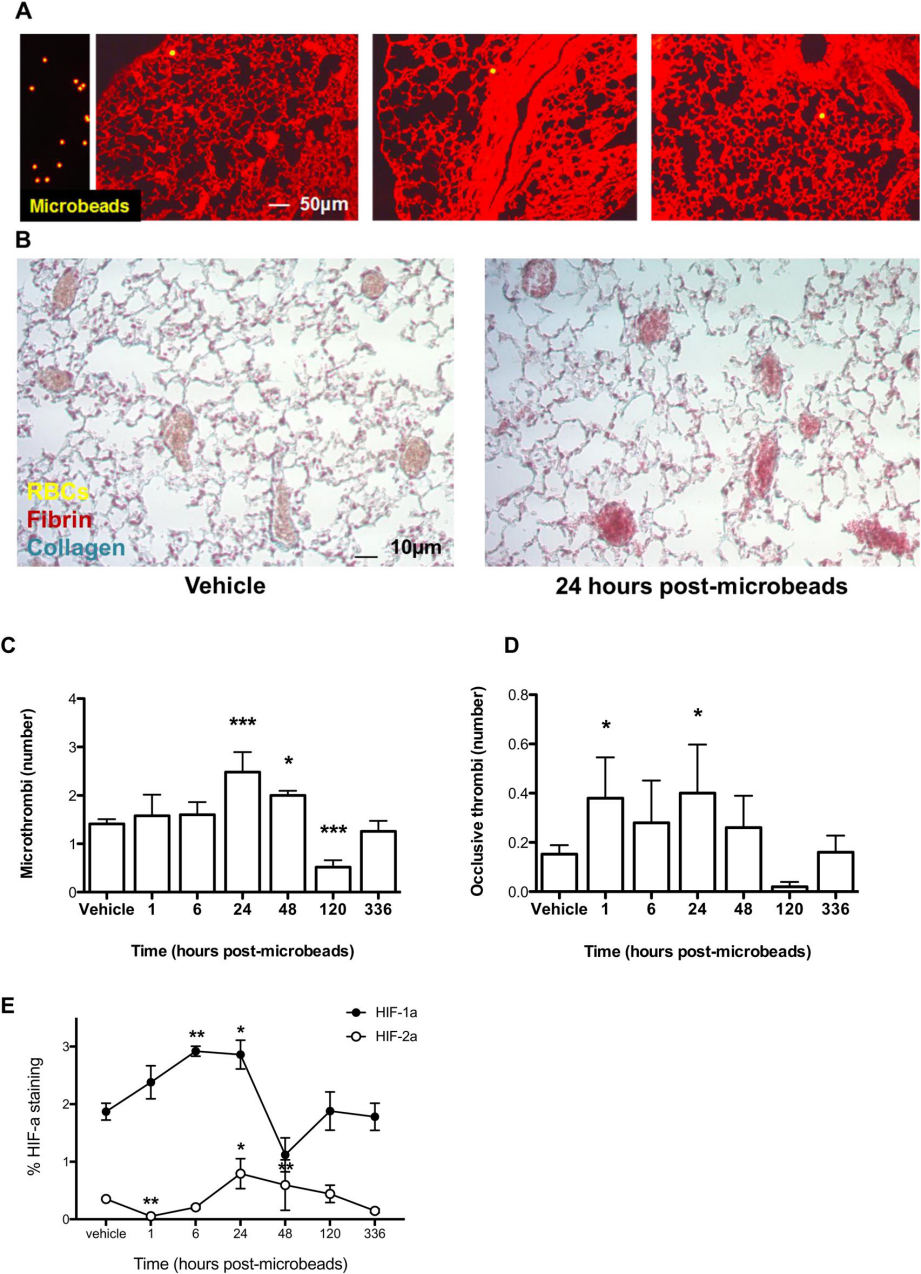
**Administration of intravenous microbeads induces pulmonary microthrombosis.** (A) Microbeads (yellow, 15 μm diameter) either isolated (left panel) or seeded intravenously into the pulmonary microvasculature at day 14 post-administration. (B) Representative lung sections were stained with MSB at day 1 post administration of intravenous microbeads or vehicle. (C) Number of total and (D) occlusive pulmonary microthrombi at various times following intravenous administration of microbeads or vehicle. (E) Pulmonary HIF1α and HIF2α levels at various times post-administration of intravenous microbeads. *N*=5/group. RBCs, red blood cells. *P<0.05 and ***P<0.001 versus vehicle-treated controls; unpaired two-tailed Student *t*-tests. Means±s.e.

Approximately 1000 microbeads were injected intravenously, a number that did not cause any discernible respiratory distress. The microbeads that seeded in the pulmonary microvasculature could be visualized by fluorescence (Fig. 1A), and ∼85% could still be detected at day 14 post-administration. This was estimated by calculating the average number of beads per cross-section and multiplying by number of cross-sections per lung. The formation of microthrombi as a result of microbead administration was assessed over time (Fig. 1B); total microthrombi numbers peaked at day 1 post-microbead injection, and returned to below baseline levels after five days (Fig. 1C). Of the microthrombi present, those that were completely occlusive were elevated as soon as one hour after injection, and resolved to levels not significantly above background after two days (Fig. 1D). This shows that the administration of the microbeads has an immediate but transient occlusive effect. We also monitored the hypoxic response to the local ischemic events by quantifying levels of pulmonary hypoxia-inducible factor (HIF)1α and HIF2α (Fig. 1E). There was a dramatic increase in HIF1α levels in the first 24 h, followed by a decrease, in a pattern that mirrors the microthrombi resolution. The stabilization of HIF2α occurred at a slower rate, and to comparatively lower levels than those observed for HIF1α, but decreased at a slower rate, suggesting distinct roles for each HIFα isoform in response to microvascular occlusion.

When pulmonary expression of both HIFα isoforms was enhanced compared to the vehicle controls (i.e. at day 1 post-treatment), regions of the pulmonary microvasculature that contained microbeads stained positively for HIF1α, HIF2α, and a macrophage cell marker, Mac2, on contiguous tissue sections (Fig. S1A). Interestingly, HIF1α- and HIF2α-positive staining was not confined to the sites of micro-occlusion, as we found increased signal for these transcription factors in regions of the pulmonary microvasculature (including lining vessel walls) that were not immediately adjacent to microbeads (Fig. S1B), suggesting a relay signal to surrounding tissues, potentially to circumvent or correct local vascular obstruction.

### Pulmonary microvascular occlusion increases pro-tumorigenic environment

**Fig. 2.**
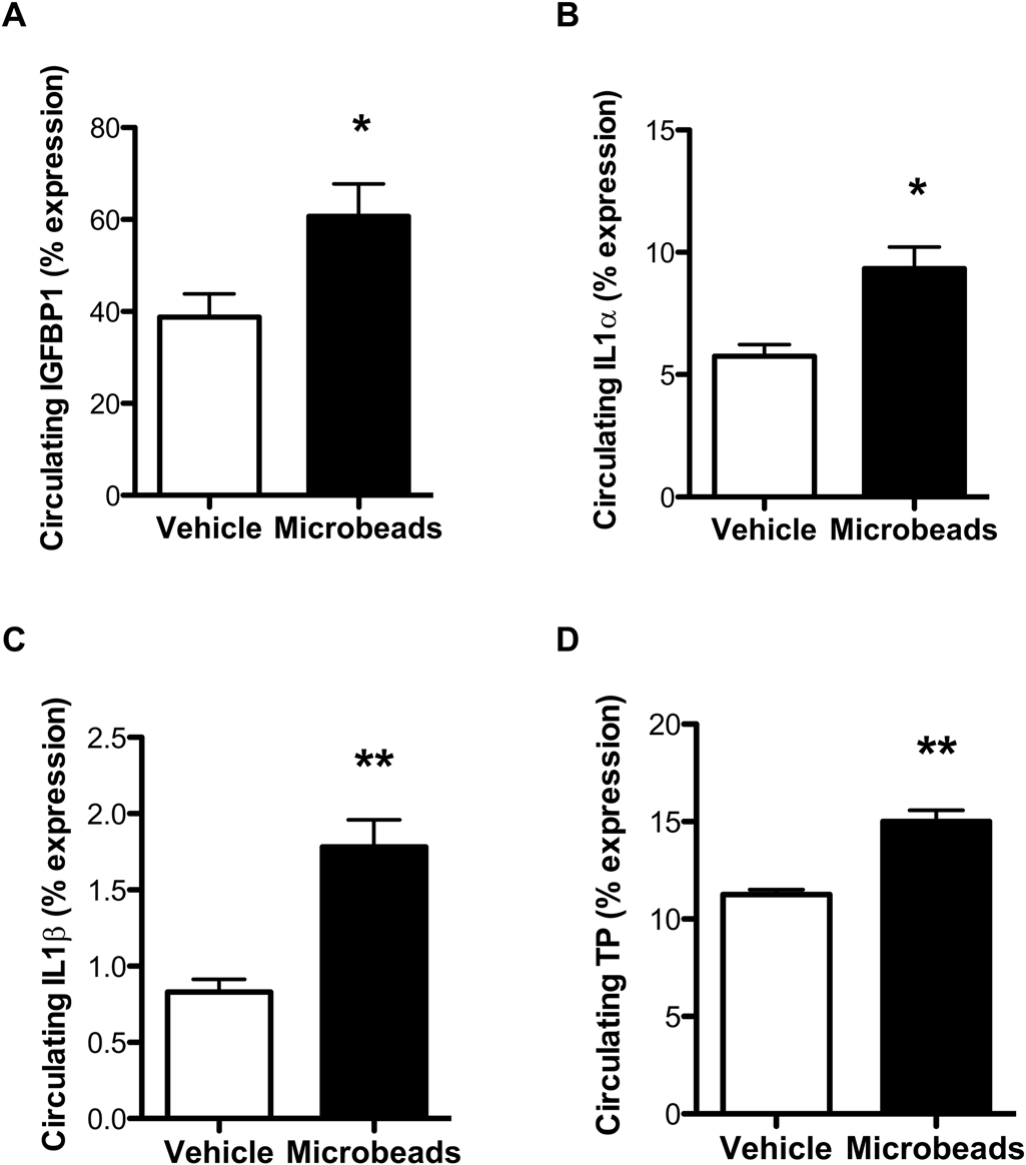
**Tumorigenic factors are increased in the circulation of microbead-treated mice.** (A-D) Quantification of tumorigenic factors IGFBP1 (A), IL1α (B), IL1β (C), and TP (D) in the circulation of mice at day 1 post-administration of intravenous vehicle or microbeads. Abbreviations are provided in Table S2. *N*=5/group. *P<0.05 and **P<0.01; unpaired two-tailed Student *t*-tests. Means±s.e.

Given that microbead administration gave rise to increases in pulmonary levels of HIF1α and HIF2α at day 1 post-treatment, we investigated whether this form of micro-occlusion could also result in enhanced local or systemic tumorigenic response. A panel of 53 tumorigenic or angiogenic factors were measured in the lungs and circulation of microbead-treated mice and their vehicle controls. Although microbead administration gave rise to modest increases in the levels of 35 (66%) of these factors in whole lung homogenates at day 1, none of these differences reached statistical significance (Table S2), likely because the obstruction is in very small regions and sampling the whole organ inevitably dilutes the signal produced at the site of occlusion. However, the levels of several tumorigenic factors detected in the circulation at day 1 post-microbead injection were significantly higher than those found in the circulation of vehicle control animals. All of these factors, i.e. insulin-like growth factor binding protein (IGFBP) 1, interleukin (IL) 1α, IL1β, and thymidine phosphorylase [TP, formerly known as platelet-derived endothelial cell growth factor (PD-ECGF)] (Fig. 2A-D) are, notably, hypoxia-responsive (Giatromanolaki et al., 2003; Nagaoka et al., 1998; Nijaguna et al., 2015; Tannahill et al., 2013). This finding reinforces the role of systemic signals in the response to even microvascular thrombotic insults.

### Pulmonary micro-occlusion increases tumour burden in a time-dependent fashion

**Fig. 3.**
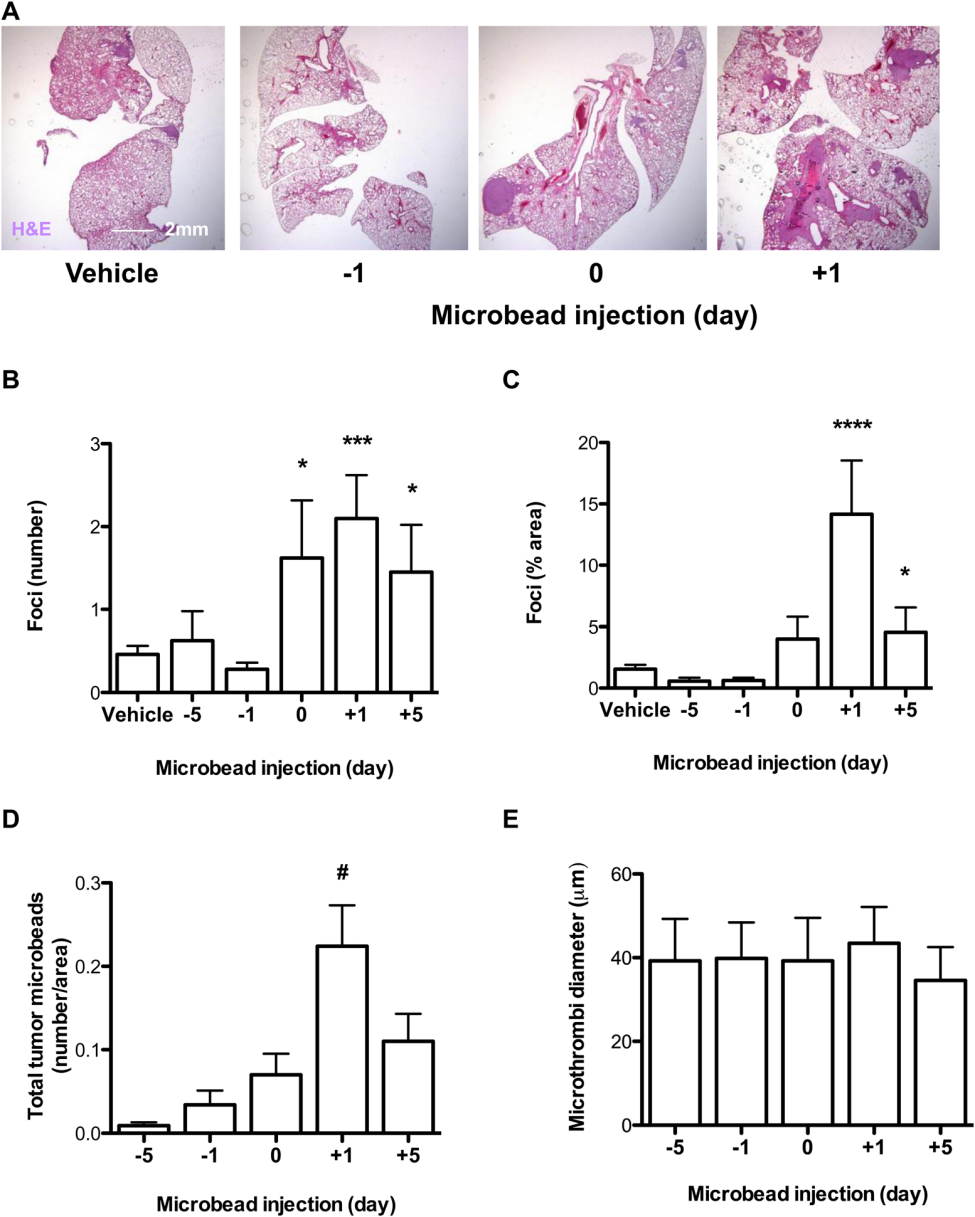
**Microbead-induced pulmonary microthrombosis increases tumour formation in a temporal manner.** (A) Representative lung sections stained with H&E following intravenous injection of vehicle or microbeads given 1 day prior (−1), at the same time (0), or 1 day after (+1) intravenous LLCs. (B) Number and (C) area of pulmonary tumours following intravenous microbead administration at different times with respect to intravenous injection of LLCs. (D) Number of tumour microbeads, and (E) microthrombi size. Mice were culled at day 14 post-LLCs. *N*=5/group. *P<0.05, ***P<0.001, and ****P<0.0001 versus vehicle-treated controls; ^#^*P*<0.05 versus days −5, −1, and 0; *, ***, and ****: unpaired two-tailed Student *t*-tests. #: ANOVA with Bonferroni post-test. Means±s.e.

To ascertain the period of time in which any pro-metastatic effect of pulmonary microvascular occlusion on tumorigenesis could occur (relative to tumour cell seeding), microbeads were introduced 1 or 5 days before, at the same time, or 1 or 5 days after tumour cell injection into the tail vein. As can be seen in Fig. 3A-C, injection of microbeads at either 1 or 5 days prior to tumour cells had no significant effect on either the number of tumour foci or the overall area of tumour tissue in lungs. However, tumour burden increased significantly when the microbeads were co-injected with tumour cells, and the maximal tumorigenic effect, both on tumour size and number, was observed when microbeads were injected the day following the tumour cells. When the microbeads were introduced 5 days after the tumour cells, there were smaller but still significant increases in tumour number and size.

**Fig. 4.**
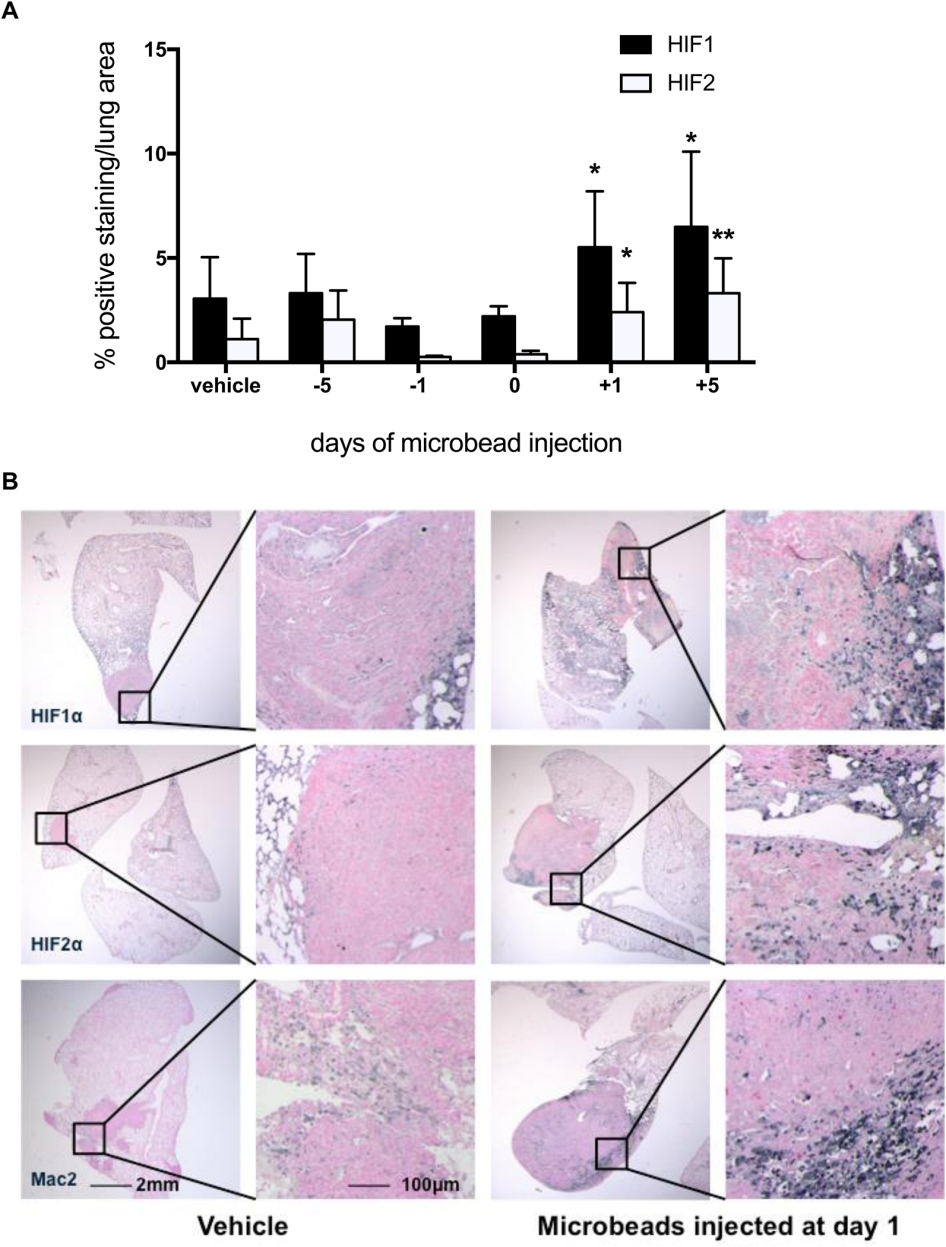
**Microbead-induced increases in pulmonary tumour formation are associated with increases in HIF1α and HIF2α expression and macrophage infiltration.** (A) Pulmonary HIF1α and HIF2α levels following microbead-induced pulmonary thrombosis in mice receiving intravenous microbeads at different times with respect to the administration of intravenous LLCs. (B) Representative lung sections stained for HIF1α, HIF2α, and myeloid cell Mac2 (dark grey/black) following intravenous administration of LLCs and either vehicle or microbeads at 1 day post-LLCs. Mice were culled at day 14 post-LLCs. *N*=5/group. *P<0.05 and **P<0.01 versus vehicle-treated controls; unpaired two-tailed Student *t*-tests. Means±s.e.

To investigate if the tumours were developing at the sites where microbeads had been arrested, we compared the number of microbeads within lung tumours between experimental groups at end-point (i.e. day 14 post-LLCs). Interestingly, we found that there were more microbeads in the tumours of mice receiving beads the day after tumour cells, when compared with those receiving beads before or concomitantly with the tumour cells (Fig. 3D), while microthrombi size (Fig. 3E), erythrocyte content (Fig. S2A), and collagen content remained similar across all treatments (Fig. S2B). Microbead-induced increases in tumour formation (i.e. those observed when beads were given after LLC injection) were accompanied by concomitant increases in both pulmonary HIF1α and pulmonary HIF2α expression (Fig. 4A,B). In these mice, strong staining for HIF1α and HIF2α was observed in regions of pulmonary tumour that also stained strongly for Mac2 (Fig. 4B).

**Fig. 5.**
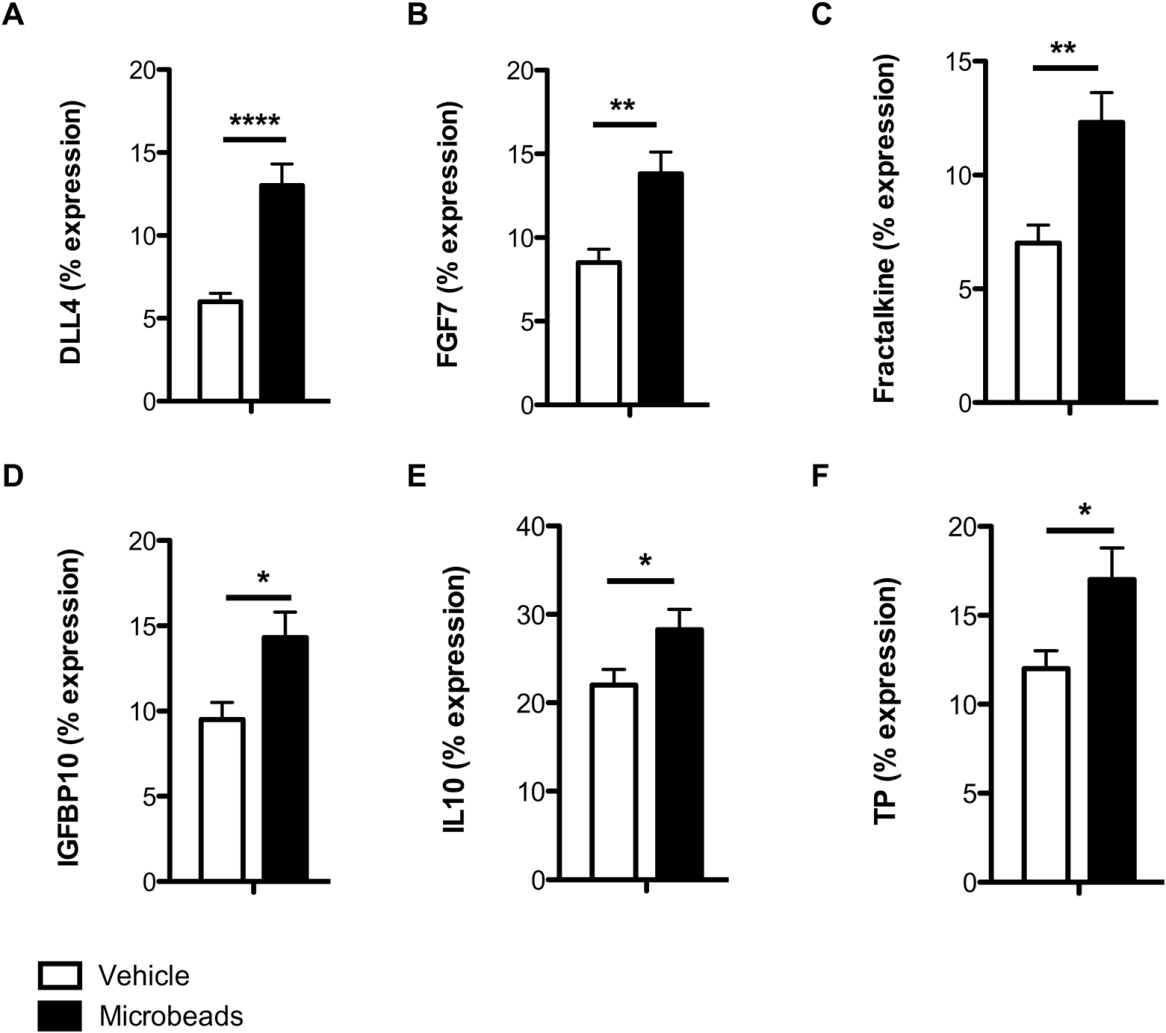
**Tumorigenic factors are increased by microbead treatment in the circulation of tumour-bearing mice.** (A-F) Quantification of DLL4 (A), FGF7 (B), fractalkine (C), IGFBP10 (D), IL10 (E), and TP (F) in the circulation of vehicle- and microbead-treated mice at day 14 post-LLC injection. Mice received intravenous microbeads at day 1 post-intravenous LLCs. Abbreviations are provided in Table S2. *N*=4-5/group. *P<0.05, **P<0.01, and ****P<0.0001; unpaired two-tailed Student *t*-tests. Means±s.e.

To determine whether the greatest increases in tumour burden could be driven by increases in circulating tumorigenic factors, the same panel of 53 tumorigenic factors described above were measured in the circulation of tumour-bearing microbead- and vehicle-treated mice on day 14 post-LLCs (endpoint). In microbead-treated mice versus vehicle controls, there were increases in the circulating levels of delta-like ligand protein (DLL) 4, fibroblast growth factor (FGF) 7, fractalkine, IGFBP10, IL10, and TP (Fig. 5A-F), all of which are involved in either angiogenesis or inflammation, processes that are essential for successful tumour establishment.

### Diverse pulmonary thrombotic insults increase tumour burden in a time-dependent fashion

During the optimization stages of this study, we also examined the effect of two other thrombotic agents on tumorigenesis. First, we substituted the microbeads with approximately 1000 fibrin microthrombi per mouse. These fibrin fragments were generated from fibrinogen activated *in vitro* and also had an approximate diameter of 15 µm (Lam et al., 2010). They also arrested in the pulmonary microvasculature and remained intact at day 14 post-administration (Fig. S3A). There were no significant effects of this form of micro-occlusion on tumorigenesis when the fibrin microthrombi injections occurred one day prior to, or at the same time as, administration of the tumour cells. However, and similarly to what was shown in the microbeads model, introduction of fibrin microthrombi 1 day after the tumour cells more than doubled the number of tumour foci detectable in the lungs, and this was accompanied by an overall doubling in the area of the lung filled by tumour mass (Fig. S3B,C).

Second, we evaluated the effect of introducing non-viable (heat-killed) tumour cells in place of the microbeads. In this case, the pro-tumorigenic impact occurred exclusively when dead and live tumour cells were injected simultaneously (Fig. S3D,E). These qualitative differences indicate that, although different types of thrombotic insults can enhance tumour formation in the lung, the nature of the agents can directly impact outcome; these discrepancies are likely in part due to the expected variability in populations of dead cells and fibrin fragments, including differential persistence in the vasculature and differential inflammatory potential.

### Correlations between thrombotic markers and HIFα in cancer patients

**Fig. 6.**
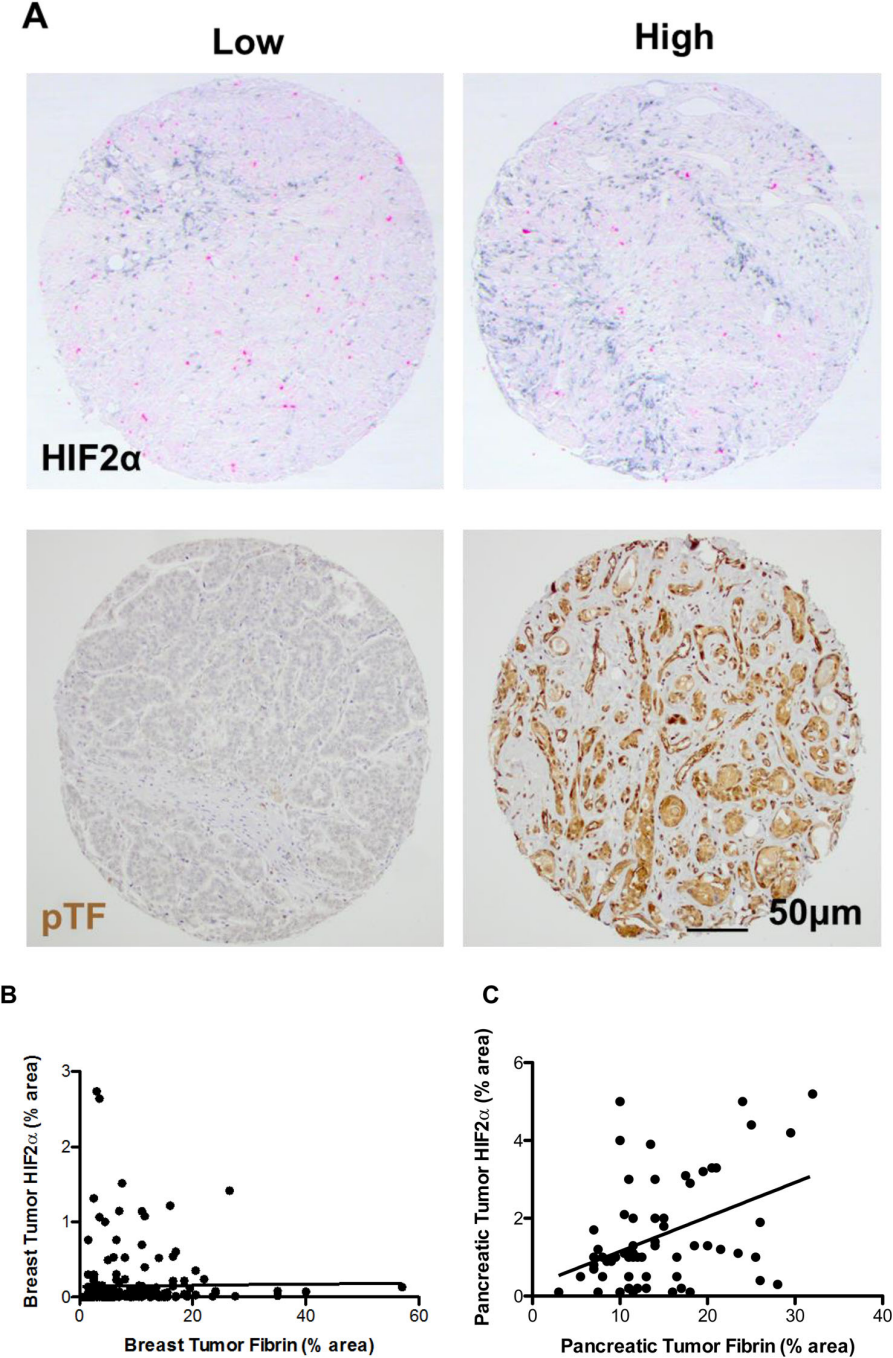
**Positive correlations between thrombotic factors and HIF2α expression in human breast and pancreatic tumours.** (A) Immunostaining for HIF2α (black) and phosphorylated tissue factor (pTF, brown) in human breast tumour microarrays. (B) Correlation between levels of HIF2α and fibrin in human breast tumours (RS=0.22; *P*<0.001) and (C) human pancreatic tumours (RS=0.33; *P*<0.01).

To determine whether thrombosis could trigger a HIF-mediated tumorigenic response in cancer patients, human breast and pancreatic tumour samples were analysed. In breast tumours, activated levels of the initiator of the coagulation cascade, phosphorylated tissue factor, were shown to be positively correlated with HIF1α expression in a recent study (Evans et al., 2016). In this study, we found that expression of phosphorylated tissue factor was also positively correlated with HIF2α expression (RS=0.2; *P*<0.005, Fig. 6A). Furthermore, we showed herein that tumour levels of the thrombotic marker plasminogen activator inhibitor 1 (PAI1), and tumour levels of the tissue factor signalling receptor protease activated receptor 2 (PAR2), were positively correlated with HIF2α but not HIF1α levels (Table 1). Finally, levels of fibrin (the terminal protein of the coagulation cascade) were also positively correlated with HIF2α (but not HIF1α) expression in breast (RS=0.22; *P*<0.001, Fig. 6B) and pancreatic tumours (RS=0.33; *P*<0.01, Fig. 6C).

**Table 1.**
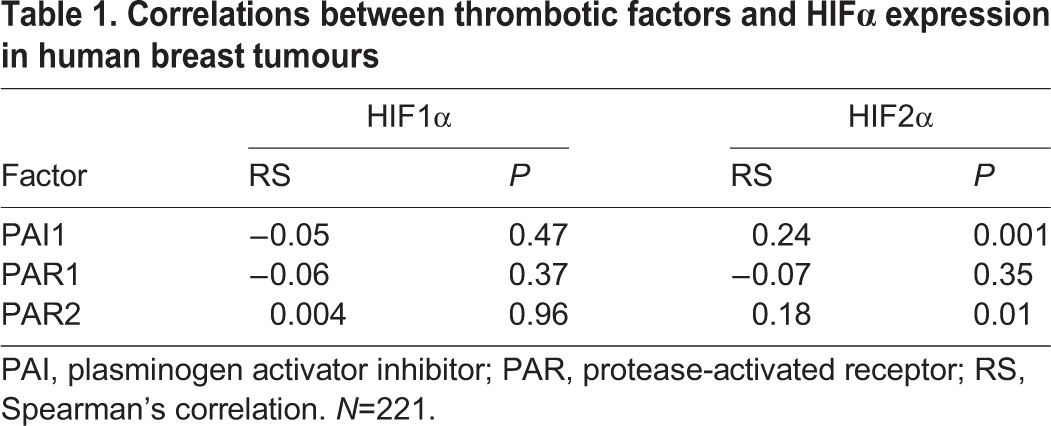
**Correlations between thrombotic factors and HIFα expression in human breast tumours**

## DISCUSSION

Hypercoagulation is often associated with exacerbated pulmonary metastasis, and it has been suggested that microthrombosis could promote metastasis by providing a scaffold for cancer cell attachment and trans-endothelial migration, a binding site for pro-metastatic growth factors and cytokines, or protection of cancer cells from natural killer cell-mediated elimination (Palumbo et al., 2005, 2007; Varki, 2007). Here we present three novel models of thrombotic occlusion coupled with metastasis in the pulmonary microvasculature, and demonstrate that these distinct insults enhance pulmonary tumour formation in a time-dependent manner. We also show that these effects depend on the nature of the obstruction.

In the current study, we present a model whereby capillary blockade by administration of sterile and inert microbeads results in the formation of obstructing and occlusive microthrombi in the lung microvasculature. These events lead to distinct temporal patterns in the activation of HIF1α and HIF2α in lungs, as well as increased infiltration of myeloid cells, chronological patterns that are comparable with those found following induction of occlusive thrombosis in an established vena cava rodent model (Evans et al., 2010, 2014c). Our findings are also consistent with observations that expression of the two major HIFα isoforms is distinct and dependent on cellular, tissue and pathological context (Elson et al., 2000; Mowat et al., 2010; Yu et al., 1998). Following thrombus formation, HIF1 activation and increased expression of proteolytic/fibrinolytic factors stimulate thrombus resolution (Evans et al., 2010), which could contribute to the reduction in microthrombi formation seen at 5 days after the introduction of occlusive microbeads to the pulmonary capillaries. Experimental studies of human pulmonary diseases modelled in murine lung could improve understanding of the mechanisms that control the distinct temporal expression patterns of the HIFα isoforms following vascular occlusion.

The finding that pulmonary tumour burden is only increased when microbead-induced pulmonary microthrombosis is given at the same time or after the introduction of circulating tumour cells is intriguing and requires further characterization in mechanistic studies. On one hand, it is possible that this effect is partly dependent upon a biomechanical influence of microvascular occlusion (Guo et al., 2014). In other words, tumour cells that arrest in the pulmonary microvasculature may be ‘pushed’ into the extra-vascular tissue by these micro-occlusive insults. This possibility is supported by the presence of microbeads at or outside of the pulmonary tumour periphery, and the attenuated effect of administering fibrin microthrombi, which are more malleable than microbeads, on pulmonary tumorigenesis. However, the fact that microbead administration results in significant increases in systemic levels of angiogenic, inflammatory and tumorigenic factors also suggests that these local ischemic insults are able to induce a response that is either secreted or diffusive. To verify whether circulating tumour cells preferentially arrest and extravasate at the site of microbead occlusion, intra-vital imaging of *in vivo* or *ex vivo* explanted lungs could be performed in future studies using different tumour cell lines (Mendoza et al., 2010).

The milder pro-metastatic effect elicited by fibrin microthrombi compared with microbeads is not unexpected, given that fibrin microthrombi may also be lysed, and can more easily be removed or extravasated from the circulation (Lam et al., 2010). Intra-venous polystyrene microbeads, however, whose number and size can be more accurately controlled, provide a relevant means of studying the effect of local vascular occlusion on the metastatic process in a model system where the occluding agent is not subject to phagocytosis or biological degradation, as may be the case with fibrin microthrombi and non-viable tumour cells. Non-viable tumour cells are also more likely to form aggregates, as well as stimulate additional and more intricate physiological responses, and the effect of circulating tumour cells (viable or not) will create an environment considerably more complex than capillary obstruction alone. For that reason, to study the effect of vascular micro-occlusion in isolation, we chose to optimise this model with an inert agent.

We show here that the systemic expression of multiple pro-tumorigenic factors is increased following microbead-induced microthrombi formation in the pulmonary microvasculature. All of these factors are hypoxia responsive and were seen to be elevated from as early as 1 day post-microbeads until as late as 14 days post-LLCs, although the distinct temporal expression patterns of individual tumorigenic factors were not fully characterized in this study. HIF target genes include many factors that exert strong pro-metastatic effects (Cramer et al., 2003; Imtiyaz and Simon, 2010). Given that pulmonary microthrombosis leads to upregulation of both HIF1α and HIF2α levels, we speculate that stabilization of these factors promotes tumorigenesis through increased secretion of pro-tumorigenic growth factors, as well as pro-inflammatory cytokines, a finding corroborated by the associated infiltration of Mac2-positive cells. This possibility is also supported by previous demonstrations that HIF1α and HIF2α are induced in occlusive and propagating venous thrombus, respectively, and are also expressed in the vessel wall adjacent to occlusive and propagating venous thrombus. Also, pharmacological upregulation of HIF1α in thrombosed mice increases the local expression of numerous pro-metastatic HIF targets, whereas inhibition of HIF1α accumulation supresses this local pro-metastatic drive (Evans et al., 2010, 2011, 2012, 2014a,b).

Although the relative contributions of cell-specific HIFs to thrombosis-associated tumorigenesis were not examined in this study, conditional tissue-specific mutant models are ideal tools for subsequent studies of such mechanisms that regulate microthrombosis-induced tumour dissemination and establishment. Future studies could investigate, for example, whether microbead-induced increases in tumour-promoting factors arise from cells within the blood, lung parenchyma, or pulmonary tumour, and whether microbead treatment gives rise to alterations in endothelial barrier permeability. The functional contribution of microthrombosis to metastatic progression could be assessed using this model in combination with approved or novel anti-coagulant pharmacotherapies.

Strong correlations between thrombotic markers and the two major HIFα isoforms have been found in cohorts of breast and pancreatic cancer patients in this and previous studies (Evans et al., 2016). These findings support the possibility that thrombotic insults could lead to increases in HIFα expression in humans (Hu et al., 2003; Zerrouqi et al., 2014). Given that primary tumours commonly metastasise to the lung, and that levels of these thrombotic markers (along with levels of HIF1α and HIF2α) accurately predict distant metastasis-free survival, we propose that thrombosis-induced increases in HIF1 and HIF2 activation could also promote metastasis in cancer patients (Bos et al., 2001, 2003; Bouchet et al., 1994; Foekens et al., 1994; Grøndahl-Hansen et al., 1993, 1997; Helczynska et al., 2008; Janicke et al., 1993; Malmstrom et al., 2001). Additional large-scale human studies of multiple cancer types are required to verify the role(s) of HIFs in thrombosis-induced tumour progression.

In summary, our novel experimental models of pulmonary microvascular occlusion demonstrate that thrombotic insults give rise to temporal increases in HIFα expression and tumour formation. Further studies of the mechanisms that regulate thrombosis-induced cancer progression could impact upon current treatment strategies for metastatic disease.

## MATERIALS AND METHODS

### Cell culture and mice

Lewis lung cancer (LLC) cells were cultured in Dulbecco's modified eagle medium with 10% fetal bovine serum and 1% penicillin/streptavidin (Life Technologies, UK) at 21% oxygen and 37°C. Studies were performed on C57BL/6J mice (8-10 week-old males, Charles River, UK) under the Animals (Scientific Procedures) Act, 1986.

### Induction of microthrombosis

For studies on the induction of pulmonary microthrombosis (*n*=5/group), fluorescent polystyrene microbeads were injected via the tail vein (15 μm diameter, 1000 beads/mouse in 100 μl saline, F8842, Life Technologies, UK). For studies on pulmonary tumour formation (*n*=5/group), polystyrene microbeads (1000/mouse in 100 μl saline), or saline vehicle (100 μl) were injected via the tail vein 1 or 5 day(s) before, at the same time, and 1 or 5 day(s) after tail vein injection of viable LLCs (1 million/mouse in 100 μl saline). During optimization stages of the protocol, fibrin microthrombi (15 μm diameter, 1000/mouse in 100 µl saline) or heat-killed tumour cells (100,000/mouse) were used as obstructive agents instead of polystyrene microbeads. Fibrin microthrombi were generated and fluorescently labelled with Texas Red (Invitrogen, UK) as described (Lam et al., 2010) and non-viable LLCs were obtained by heat killing at 60°C for 30 min.

### Histology and immunostaining

Mice were culled at indicated time points post-microbeads' administration or 14 days post-LLC injection as described (Branco-Price et al., 2012). Lungs were fixed in 4% paraformaldehyde and embedded in paraffin and cross-sections (7 μm) were cut at 200 μm intervals throughout the lung. Contiguous sections were stained with haematoxylin and eosin (H&E, Sigma Aldrich, UK), Martius Scarlet Blue (MSB, Sigma Aldrich, UK), and for HIF1α (Novus Biologicals, UK), HIF2α (Novus Biologicals, UK), and Mac2 (BioLegend Ltd, UK) as previously described (Evans et al., 2010, 2014a,b,c; Saha et al., 2013).

### Image capture and analysis

Images were captured in a blinded fashion using a light or confocal microscope and mounted camera (Leica, UK). Microthrombi numbers were assessed by counting fibrin-positive (red) deposits in the microvascular lumen of MSB-stained lung tissue as described (Evans et al., 2016). Occlusive microthrombi were defined as those that completely filled the vessel lumen. Fibrin, collagen, and erythrocyte content, and primary antibody binding (for HIF1α, HIF2α, and Mac2) were assessed by quantification of positive staining using Image J (NIH, USA) as described (Evans et al., 2010, 2014a; Saha et al., 2013). Regions of positive staining were delineated and expressed as a percentage of tissue cross-sectional area. The assessment of fibrin deposition by quantification of the red regions of MSB-stained tissue has been previously validated in an established murine thrombosis model; i.e. fibrin levels assessed by quantification of MSB-stained tissue correlate strongly with fibrin levels derived by both fibrin immunostaining and western blotting (Saha et al., 2013). Tumour burden was assessed as described (Branco-Price et al., 2012). Number of microbeads in contact with tumour foci were counted and expressed per surface area of tumour tissue by delineation of the tumour boundary using Image J (NIH, USA). For all variables, an average was taken from three or more whole lung cross-sections taken at 200 µm intervals throughout the entire tissue.

### Protein arrays

A panel of 53 tumorigenic growth factors and cytokines were measured in the circulation and lungs of vehicle- and microbead-treated mice at day 1 post-microbeads (*n*=5/group) and in the circulation of tumour-bearing mice at day 14 post-LLCs (*n*=4-5/group) by protein array according to manufacturers' instructions (Proteome Profiler Array, ARY015, R&D Systems, UK). Serum and lung samples were also prepared according to the manufacturers' instructions of the array.

### Human tumour microarrays

Human studies were approved by the ethics committee of Lund University Hospital and were performed with informed consent from all subjects. Breast tumour microarray samples and plasma were obtained from a lymph node-negative population of breast cancer patients that have previously been described (*n*=221) (Malmstrom et al., 2001). Pancreatic tumour microarray samples were obtained from the Human Tissue Bank at NIHR Cambridge Biomedical Research Centre (*n*=63, Table S1). In breast tumours, levels of protease-activated receptor 1 and 2 were quantified by immunostaining and image analysis as described (Ryden et al., 2010), and plasminogen activator inhibitor 1 was quantified by enzyme-linked immunosorbent assay (ELISA) as described (Linderholm et al., 2008; Malmstrom et al., 2001). In breast and pancreatic tumours, fibrin content, and HIF1α and HIF2α expression were assessed by image analysis of stained tissue as described above.

### Statistical analysis

Pairwise comparisons were analyzed by the unpaired two-tailed Student *t*-test. ANOVA with Bonferroni post-test was used to test differences between multiple groups. Correlations were assessed using Spearman's correlation. Significance was assumed at *P*<0.05 in all analyses (Prism 5, Graphpad, USA). Data are expressed as means±standard error.
